# Serum Hepcidin and Soluble Transferrin Receptor in the Assessment of Iron Metabolism in Children on a Vegetarian Diet

**DOI:** 10.1007/s12011-017-1003-5

**Published:** 2017-03-24

**Authors:** Jadwiga Ambroszkiewicz, Witold Klemarczyk, Joanna Mazur, Joanna Gajewska, Grażyna Rowicka, Małgorzata Strucińska, Magdalena Chełchowska

**Affiliations:** 1 Screening Department, Institute of Mother and Child, Kasprzaka 17A, 01-211 Warsaw, Poland; 2 Department of Nutrition, Institute of Mother and Child, Kasprzaka 17A, 01-211 Warsaw, Poland; 3 Department of Child and Adolescent Health, Institute of Mother and Child, Kasprzaka 17A, 01-211 Warsaw, Poland

**Keywords:** Hepcidin, Soluble transferin receptor, vegetarian diet, Children

## Abstract

The aim of this study was to assess the effect of vegetarian diet on iron metabolism parameters paying special attention to serum hepcidin and soluble transferrin receptor (sTfR) concentrations in 43 prepubertal children (age range 4.5–9.0 years) on vegetarian and in 46 children on omnivorous diets. There were no significant differences according to age, weight, height, and body mass index (BMI) between vegetarian and omnivorous children. Vegetarians had similar intake of iron and vitamin B_12_ and a significantly higher intake of vitamin C (*p* < 0.05) compared with non-vegetarians. Hematologic parameters and serum iron concentrations were within the reference range in both groups of children. Serum transferrin levels were similar in all subjects; however, ferritin concentrations were significantly (*p* < 0.01) lower in vegetarians than in omnivores. In children on a vegetarian diet, median hepcidin levels were lower (*p* < 0.05) but sTfR concentrations significantly higher (*p* < 0.001) compared with omnivorous children. In the multivariate regression model, we observed associations between hepcidin level and ferritin concentration (*β* = 0.241, *p* = 0.05) in the whole group of children as well as between hepcidin concentration and CRP level (*β* = 0.419, *p* = 0.047) in vegetarians. We did not find significant associations with concentration of sTfR and selected biochemical, anthropometric, and dietary parameters in any of the studied groups of children. As hematologic parameters and iron concentrations in vegetarians and omnivores were comparable and ferritin level was lower in vegetarians, we suggest that inclusion of novel markers, in particular sTfR (not cofounded by inflammation) and hepcidin, can better detect subclinical iron deficiency in children following vegetarian diets.

## Introduction

Iron deficiency in children has been associated with negative effects on cognitive and motor development and with behavioral problems [1–3]. Although a range of hematologic and biochemical iron status indicators have been described in the past, there is a consensus that no single blood test adequately reflects iron status in the body. The usual markers might not be able to adequately distinguish at subclinical levels; therefore, much attention was paid to the search of novel markers involved in the regulation of iron metabolism.

Hepcidin is a small cysteine-rich 25 amino acid antimicrobial peptide that plays a central role in iron homeostasis [4–6]. The hepatic cells are a major site of hepcidin production, but to some extent, hepcidin messenger RNA (mRNA) is also expressed in other tissues. Hepcidin negatively regulates iron availability by binding and degrading ferroportin, a transmembrane iron transporter necessary for iron transfer out of intestinal epithelial cells and macrophages [5, 7]. Low levels of hepcidin enable increased iron absorption from dietary sources and its mobilization from hepatocytes and macrophages, while elevated hepcidin levels inhibit this pathway. Production of hepcidin is induced by iron overload and inflammation. Gene expression of this hormone is downregulated by anemia, hypoxia, erythropoietic activity, and iron deficiency [5, 7, 8]*.*

Serum transferrin receptor (sTfR) is a soluble form of the membrane receptor derived from its proteolysis. Transferrin receptor expression on the cell surface and soluble transferrin receptor concentrations are negatively correlated with intracellular iron levels [6]. It seems that sTfR level is a better marker of iron status compared with other parameters, because its concentration is less affected by inflammation but could be affected by erythropoietic activity [9]. It appears that serum hepcidin and sTfR might provide more information than ferritin and hematologic parameters in assessing iron status*.*

Since the health benefits of vegetarian diets were reported, vegetarianism has been gaining popularity among families as a lifestyle choice [10–12]. In general, the vegetarian dietary pattern, if well balanced, can be adequate for adults and children [13–15]. However, certain components of these diets and some required nutrients (including iron) need specific attention in growing children and adolescents. A very small number of studies based on a small cohort of patients have been carried out on iron markers and hematologic status in vegetarian children [16–18].

The aim of this study was to assess iron metabolism parameters paying special attention to serum hepcidin and soluble transferrin receptor concentrations in prepubertal children following a lacto-ovo vegetarian diet and traditional diet. Furthermore, we analyzed the association between levels of hepcidin as well as sTfR and other iron status markers.

## Materials and Methods

### Patients

The study was conducted according to the principles of the Declaration of Helsinki and was approved by the Ethics Committee of the Institute of Mother and Child. Written informed consent was obtained from all parents of the children before their participation in the study.

Eighty-nine prepubertal children (age range 4.5–9.0 years) were recruited between January 2015 and July 2016 from a group of consecutive patients seeking dietary counseling in the Department of Nutrition at the Institute of Mother and Child in Warsaw (Poland). Pubertal stage was assessed according to the Tanner criteria. All participants were Caucasian. None of the subjects had any serious health problems nor were receiving any medications. Children whose clinical characteristics could have affected iron metabolism, inflammation, altered liver function, or eating disorders were excluded. Also, children with gastrointestinal disorders such as lactose intolerance, celiac disease, irritable bowel syndrome, or constipation were excluded. At the Institute of Mother and Child, consultations with medical and nutritional specialists are available to children on a vegetarian as well as on a traditional mixed diet, whose parents want to make sure that their children are properly nourished. The study group consisted of 43 children on a lacto-ovo vegetarian diet, who did not consume meat, poultry, or fish, but ate eggs and dairy products. The parents of these children were vegetarians and the children were following a lacto-ovo vegetarian diet from birth. The omnivorous group included 46 healthy prepubertal children on a traditional mixed diet who had visited the Institute of Mother and Child for routine medical checkups and dietary consultation.

## Methods

### Assessment of Dietary Intake

Dietary constituent data were assessed by questionnaire and calculated using the nutritional computer program Dieta5® (National Food and Nutrition Institute, Warsaw). We assessed dietary intakes using Diet Record methods. Before medical consultation, the parents were asked to prepare a 10-day food diary for their children. They had been previously advised by the nutritionist on how to do it correctly. In the Department of Nutrition, the diary was checked by the nutritionist in the presence of child and parent. The nutritionist asked for detailed information about the foods and drinks recorded, such as portion sizes and preparation methods using photo album of products and dishes. When necessary, the food diary was corrected by the nutritionist during the visit. Out of the 10 days in the record, three consecutive days (two weekdays and one weekend day) were selected. These data were entered into a nutritional software program for analysis to evaluate average daily energy intake, the percentage of energy from protein, fat, and carbohydrates, and dietary iron and vitamin intakes in the children’s diets. The obtained results were compared to the current recommendations for supplying essential nutrients for the Polish population; the age- and sex-specific dietary reference intake (DRI) percentage was calculated using reference values of daily energy intake for children according to Jarosz et al. [19]. Our studied children because of the age range (4.5–9.0 years) were among the two groups mentioned in the above recommendations (group 4–6 years and group 7–9 years). The data for each child was compared to the recommendations for appropriate age and gender.

### Anthropometric Measurements

Anthropometric measurements such as height and weight were performed in all children. Height was measured with a stadiometer and recorded with a precision of 0.1 cm, and weight was assessed unclothed with a calibrated balance scale to the nearest 0.1 kg. Body mass index (BMI) was calculated as body weight (kg) divided by height squared (m^2^).

### Hematologic and Biochemical Analyses

Fasting peripheral blood samples (3 mL) were taken in the morning hours (8–10 a.m.) for all children. Blood in EDTA-containing tubes was analyzed immediately for the determination of hemoglobin (Hb), red blood cells (RBC), and mean corpuscular volume (MCV) using a hematology analyzer (HORRIBA ABX, France). To obtain serum, the blood was centrifuged at 2500×*g*, at 4 °C for 10 min, and stored in small portions at −25 °C for subsequent biochemical analysis. Serum iron, ferritin, and transferrin concentrations as well as C-reactive protein (CRP) as an inflammatory marker were determined using commercially available kits on biochemical analyzer (ROCHE, Switzerland). Serum hepcidin (bioactive heptidin-25 molecule) and soluble transferrin receptor concentrations were measured using commercially available ELISA kits (DRG, Germany). According to the test principle, serum samples were incubated with monoclonal anti-hepcidin-25/anti-human sTfR antibody, then conjugated with proper enzyme complex, proper substrate solution, and finally the absorbance, which is proportional to the concentration of hepcidin-25/sTfR in the patient’s serum sample, was measured. The detection limit was 0.153 ng/mL for hepcidin and 0.02 mg/L for sTfR. The intra- and inter-assay coefficients of variation (CVs) were less than 5.7 and 9.5% for hepcidin and 6.0 and 7.0% for sTfR, respectively.

### Statistical Analyses

All statistical analyses were performed using SPSS statistical software version 17.1 (SPSS INC., Chicago, IL, USA). The normality of data was tested using the Kolmogorov-Smirnov test. The results were presented as means and standard deviation (SD) for normally distributed data or median and interquartile range (25th–75th percentiles) for non-normally distributed variables. In vegetarian and omnivorous children, the baseline characteristics were compared using Student’s *t* test or the Mann-Whitney *U* test depending on the assumptions. The chi-squared test was used for comparing categorical variables. Univariate and multivariate regression models with hepcidin and sTfR concentrations as dependent variables were assessed to examine the potential impact of the clinical, anthropometric, dietary, hematological, and biochemical variables. Forced entry method was used to show relationships between iron status markers and hepcidin as well as sTfR. The results were presented as the value of β standardized regression coefficient, B unstandardized regression coefficient with 95% confidence interval (CI), and change in R-squared coefficient of determination after each variable was entered. The models were estimated separately for the whole group of children, as well as for vegetarian and omnivore subgroups. In multivariate models, analysis of collinearity was conducted with two standard statistics of tolerance and VIF (variance inflation factor). The differences were regarded as statistically significant at *p* < 0.05.

## Results

Average daily energy and macronutrient intake were within recommended daily intake; however, the proportions of proteins and carbohydrates in vegetarian and omnivorous diets differed (Table 1). The percentage of energy from protein was significantly lower (*p* < 0.05) and energy from carbohydrates higher (*p* < 0.01) in vegetarians than omnivores. The vegetarian diet had a similar intake of iron and vitamin B_12_ and a significantly higher intake of vitamin C (*p* < 0.05) than the omnivorous diet. Analyzing the age- and sex-specific DRI percentage in the vegetarian group, 91.4% of children realized dietary age- and sex-specific dietary reference intake for energy, with 97.7% protein, 90.7% fat, and 99.6% carbohydrates.

**Table 1:** Daily energy and nutrient intake of the examined children compared with recommended daily intake

	Vegetarian children	Omnivorous children	*p*	Recommended daily intake^d^
Energy (kcal/day)^a^	1443.4 ± 360.5	1551.5 ± 434.4	0.391	4–6 years, 1400; 7–9 years, 1800–2100
Protein (g/day)^a^	42.5 ± 14.5	51.2 ± 14.9	0.057	4–6 years, 21; 7–9 years, 30–42
Energy from protein (%)^c^	12.4 ± 2.0	13.7 ± 2.4	0.030	4–18 years, 10–30
Fat (g/day)^a^	53.2 ± 20.3	60.1 ± 25.9	0.329	4–6 years, 31–54; 7–9 years, 40–70
Energy from fat (%)^c^	30.2 ± 6.3	34.8 ± 8.6	0.100	4–18 years, 50–70
Carbohydrates (g/day)^a^	223.9 ± 62.2	208.8 ± 49.6	0.495	4–6 years, 130; 7–9 years, 130
Energy from carbohydrates (%)^c^	57.4 ± 6.6	51.5 ± 8.4	0.001	4–18 years, 50–70
Iron (mg/day)^a^	9.5 ± 3.5	8.9 ± 2.1	0.150	4–6 years, 10; 7–9 years, 10
Vitamin C (mg/day)^b^	85 (38–143)	60 (37–90)	0.015	4–6 years, 50; 7–9 years, 50
Vitamin B_12_ (μg/day)^b^	1.7 (1.1–2.3)	1.8 (1.4–3.0)	0.134	4–6 years, 1.2; 7–9 years, 1.8

^a^Data are presented as mean values ± standard deviation (SD)

^b^Data are presented as median values and interquartile ranges (25th–75th)

^c^Data are presented as percentage

^d^Data are presented as recommended daily energy and nutrient intakes according to Jarosz et al. [19]

Clinical, anthropometric, hematologic, and biochemical variables of the studied children are presented in Table 2. There were no significant differences according to age, weight, height, or BMI between vegetarian and omnivorous children. The median level of CRP (marker of inflammation) was lower in vegetarian children than omnivores, but CRP values were within the normal range in all individuals. There were no signs of anemia in the studied children, who presented normal hemoglobin, RBC, and MCV, except for 1 vegetarian and 1 control subject who had hemoglobin below 11.5 g/mL. About 9% of children (4 vegetarians and 4 omnivores) had decreased MCV (<80 fL); however, these values were in the lower range limit of the norm (78–79 f/L). Red blood cells were within the reference range in all subjects. There was no significant difference in the mean value of serum iron between vegetarians and omnivores. Despite this, 11 (25.6%) vegetarians and 7 (15.2%) omnivores had decreased iron concentration. Serum transferrin levels were similar in both groups of children; however, ferritin concentrations were significantly (*p* < 0.01) lower in vegetarians. Among them, 10 (23.3%) vegetarians and 2 (4.6%) omnivores had ferritin below 15 ng/mL. In children on a vegetarian diet, median hepcidin levels were significantly lower (about twofold, *p* < 0.05) but sTfR concentration significantly higher (*p* < 0.001) compared with omnivorous children.

**Table 2:** Clinical and anthropometric data, hematologic, and biochemical parameters in vegetarian and omnivorous children

	Vegetarian children	Omnivorous children	*p*
Age (year)^b^	6.5 (5.5–8.7)	6.3 (5.0–8.0)	0.307
Sex, female/male (%)^c^	58.1/41.9	41.3/58.7	0.084
Weight (kg)^a^	23.4 ± 7.4	20.9 ± 6.1	0.088
Height (m)^a^	1.22 ± 1.62	1.18 ± 1.27	0.129
BMI (kg/m^2^)^a^	15.24 ± 1.21	14.86 ± 1.39	0.159
Hepcidin (ng/mL)^b^	5.46 (3.63–12.92)	11.54 (7.00–15.15)	0.012
sTfR (mg/L)^a^	1.33 ± 0.30	1.12 ± 0.26	0.001
Iron (μmol/L)^a^	15.68 ± 6.47	15.28 ± 5.87	0.762
Ferritin (ng/mL)^b^	21.0 (15.0–29.0)	27.0 (21.6–48.7)	0.003
Transferrin (mg/dL)^a^	295.7 ± 31.2	299.1 ± 33.8	0.748
Hb (g/dL)^a^	12.65 ± 1.93	12.91 ± 0.74	0.394
MCV (fL)^a^	83.97 ± 4.03	82.68 ± 4.09	0.139
RBC (×10^6^/μL)^a^	4.52 ± 0.29	4.62 ± 0.28	0.101
CRP (mg/L)^b^	0.14 (0.08–0.25)	0.31 (0.09–0.74)	0.011

*CRP* C-reactive protein, *BMI* body mass index, *sTfR* soluble transferrin receptor, *Hb* hemoglobin, *MCV* mean corpuscular volume, *RBC* red blood cells

^a^Data are presented as mean values ± SD

^b^Data are presented as median values and interquartile ranges (25th–75th)

^c^Data are presented as percentage

In univariate regression analyses defined for the whole group of children, we found that serum hepcidin level was associated with ferritin (*B* = 0.179, *p* = 0.025) and CRP (*B* = 2.766, *p* = 0.012) concentrations (Table 3). In the model estimated separately for vegetarians, we showed relations between concentrations of hepcidin and CRP (*B* = 5.866, *p* = 0.001) and between hepcidin levels and dietary iron (*B* = 1.539, *p* = 0.045) and protein (*B* = 0.267, *p* = 0.021) intakes. However, we did not observe correlations between hepcidin concentration and iron status markers, anthropometric parameters, as well as dietary variables in omnivores. As shown in Table 4, significant predictor of sTfR level was ferritin (*B* = −0.004, *p* = 0.041) in the whole group of children and hemoglobin and RBC (*B* = 0.589, *p* = 0.044; *B* = 0.340, *p* = 0.012, respectively) in omnivorous group. We did not find such correlations in vegetarians.

**Table 3:** Univariate regression analyses of hepcidin with clinical and nutritional data and iron status parameters in the whole group of studied children (*n* = 89) and in the subgroups of vegetarian children (*n* = 43) and omnivorous children (*n* = 46)

	All children			Vegetarian children			Omnivorous children		
	*B*	95% CI	*p*	*B*	95% CI	*p*	*B*	95% CI	*p*
Age Sex	−0.256 −1.507	−1.361/0.849 −6.139/3.125	0.646 0.519	−0.125 0.378	−1.629/1.379 −6.801/7.557	0.867 0.916	−0.283 −4.290	−2.036/1.471 −10.569/0.175	0.747 0.175
BMI Dietary protein Dietary fat Dietary carbohydrates Dietary iron Dietary vitamin C Dietary vitamin B_12_	−0.823 0.149 0.015 −0.005 0.665 0.025 −0.090	−2.633/0.988 −0.017/0.315 −0.088/0.117 −0.057/0.047 −0.224/1.554 −0.030/0.080 −2.059/1.879	0.369 0.078 0.777 0.852 0.141 0.364 0.928	−1.103 0.267 0.005 −0.051 1.539 0.017 0.154	−4.035/1.829 0.043/0.491 −0.143/0.153 −0.129/0.026 0.039/3.038 −0.111/0.145 −2.491/2.799	0.452 0.021 0.949 0.190 0.045 0.789 0.907	−0.343 0.054 0.050 0.038 0.058 0.017 0.062	−2.738/0.774 −0.209/0.317 −0.100/0.200 −0.031/0.107 −1.062/1.177 −0.046/0.081 −3.215/3.339	0.774 0.680 0.506 0.276 0.918 0.587 0.970
Hemoglobin	0.811	−0.798/2.421	0.319	0.725	−1.118/2.567	0.432	0.760	−3.529/5.050	0.723
RBC	6.465	−1.480/14.419	0.110	9.010	−2.960/20.980	0.136	2.599	−8.707/13.905	0.645
MCV	0.198	−0.371/0.768	0.491	0.288	−0.598/1.173	0.515	0.232	−0.545/1.009	0.550
Serum iron	−0.072	−0.453/0.308	0.706	0.159	−0.393/0.711	0.563	−0.319	−0.854/0.216	0.236
Ferritin	0.179	0.023/0.336	0.025	0.226	−0.090/0.543	0.157	0.138	−0.059/0.335	0.165
sTfR	−0.080	−7.920/7.759	0.984	−2.382	−14.288/9.525	0.688	6.917	−5.150/18.984	0.254
CRP	2.766	0.613/4.919	0.012	5.866	2.429/9.303	0.001	0.642	−2.157/3.442	0.646

*BMI* body mass index, *RBC* red blood cells, *MCV* mean corpuscular volume, *sTfR* soluble transferrin receptor, *CRP* C-reactive protein

**Table 4:** Univariate regression analyses of sTfR with clinical and nutritional data and iron status parameters in the whole group of studied children (*n* = 89) and in the subgroups of vegetarian children (*n* = 43) and omnivorous children (*n* = 46)

	All children			Vegetarian children			Omnivorous children		
	*B*	95% CI	*p*	*B*	95% CI	*p*	*B*	95% CI	*p*
Age Sex	−0.006 −0.028	−0.036/0.024 −0.154/0.098	0.698 0.655	−0.029 0.160	−0.067/0.010 −0.023/0.343	0.142 0.085	0.016 −0.137	−0.027/0.059 −0.291/0.016	0.458 0.079
BMI Dietary protein Dietary fat Dietary carbohydrates Dietary iron Dietary vitamin C Dietary vitamin B_12_	0.014 0.000 0.000 0.001 −0.018 0.000 0.037	−0.035/0.064 −0.004/0.005 −0.002/0.003 −0.001/0.002 −0.042/0.006 −0.001/0.002 −0.015/0.090	0.565 0.915 0.782 0.392 0.138 0.716 0.163	0.000 −0.004 0.000 0.000 −0.006 0.002 0.030	−0.078/0.078 −0.010/0.003 −0.004/0.004 −0.002/0.002 −0.047/0.036 −0.001/0.006 −0.039/0.100	0.992 0.237 0.943 0.879 0.783 0.156 0.382	0.004 0.002 −0.001 0.002 −0.018 0.001 0.009	−0.056/0.063 −0.005/0.008 −0.004/0.003 0.000/0.003 −0.045/0.009 −0.001/0.002 −0.073/0.090	0.904 0.597 0.739 0.063 0.184 0.374 0.832
Hemoglobin	−0.004	−0.048/0.040	0.868	−0.013	−0.062/0.036	0.589	0.105	0.003/0.206	0.044
RBC	0.096	−0.123/0.314	0.385	−0.012	−0.338/0.313	0.939	0.340	0.079/0.601	0.012
MCV	-0.002	−0.017/0.014	0.818	−0.001	−0.025/0.022	0.901	−0.010	−0.029/0.009	0.294
Serum iron	−0.004	−0.014/0.007	0.484	−0.006	−0.020/0.009	0.447	−0.003	−0.016/0.011	0.688
Ferritin	−0.004	−0.009/0.000	0.041	−0.001	−0.010/0.007	0.730	−0.003	−0.008/0.002	0.272
Hepcidin	0.000	−0.006/0.006	0.984	−0.002	−0.010/0.007	0.688	−0.004	−0.003/0.012	0.254
CRP	-0.025	−0.085/0.036	0.418	−0.005	−0.108/0.098	0.926	−0.011	−0.080/0.059	0.757

*BMI* body mass index, *RBC* red blood cells, *MCV* mean corpuscular volume, *sTfR* soluble transferrin receptor, *CRP* C-reactive protein

In the multivariate regression model, we observed associations between hepcidin and ferritin concentrations (*β* = 0.241, *p* = 0.05) in the whole group of children, between hepcidin and CRP levels (*β* = 0.419, *p* = 0.047) in vegetarians, but we did not find such correlations in omnivores (Table 5). R-squared (expressed as percentage of a variation that can be explained by a linear regression model) was 22.6% for the whole group of children, 18.4% for the group of omnivorous children, and 45.0% for vegetarian children. Effect of collinearity that could affect the results was not observed. Multivariate regression analysis did not confirm the impact of selected biochemical, anthropometric, and dietary parameters on the level of sTfR in any of the studied groups of children.

**Table 5:** Multivariate regression of hepcidin and sTfR with iron status markers, inflammation marker and some dietary parameters in the whole group (*n* = 89, adjusted for age, sex, BMI, dietary status) of studied children and vegetarian (*n* = 43, adjusted for age, sex, BMI) and omnivore (*n* = 46) subgroups (adjusted for age, sex, BMI)

	Hepcidin						sTfR					
	All children		Vegetarians		Omnivores		All children		Vegetarians		Omnivores	
	*β*	*p*	*β*	*p*	*β*	*p*	*β*	*p*	*β*	*p*	*β*	*p*
Hemoglobin	−0.003	0.830	−0.152	0.435	−0.027	0.952	−0.045	0.750	−0.151	0.527	0.713	0.067
RBC	0.237	0.109	0.385	0.070	0.139	0.716	0.201	0.182	0.231	0.383	−0.312	0.360
MCV	0.243	0.099	0.442	0.074	0.162	0.636	0.063	0.679	0.220	0.476	−0.485	0.107
Serum iron	0.060	0.617	0.213	0.289	−0.123	0.501	−0.117	0.334	−0.099	0.690	−0.213	0.191
Ferritin	0.241	0.050	0.192	0.216	0.296	0.138	−0.135	0.286	−0.032	0.866	−0.193	0.285
Hepcidin	–	–	–	–	–	–	0.080	0.499	−0.028	0.899	0.138	0.376
sTfR	0.077	0.499	−0.019	0.899	0.173	0.376	–	–	–	–	–	–
CRP	0.165	0.168	0.419	0.047	−0.011	0.952	−0.036	0.766	0.004	0.987	−0.080	0.613
Dietary protein	0.038	0.758	0.093	0.612	0.006	0.975	−0.070	0.565	−0.097	0.678	0.072	0.668
Dietary iron	0.173	0.143	0.052	0.785	0.124	0.511	−0.102	0.411	−0.133	0.549	−0.058	0.954
*R* ^2^ (%)	22.6		45.0		18.4		20.2		18.5		34.7	

*RBC* red blood cells, *MCV* mean corpuscular volume, *sTfR* soluble transferrin receptor, *CRP* C-reactive protein

## Discussion

The statements of the American Dietetic Association and Dietitians of Canada, as well as the results of several clinical studies, consider that a well-planned vegetarian diet is adequate for all stages of the life cycle, including childhood [20–22]. However, this kind of diet should be under medical and dietetic control in young children, who have high physiologic requirements for iron to support their rapid growth and brain development. The data from industrialized countries showed that for children on vegetarian diets, iron intakes are similar or even higher than those of omnivores [16, 21, 23, 24]. Similarly, in our study, vegetarian and omnivorous diets had comparable iron intakes. In vegetarian diets, the bioavailability of iron is poor because of nonheme iron from plant foods. Additionally, vegetarian diets contain high amounts of iron absorption inhibitors (polyphenols and phytate), which form insoluble complexes and cannot be digested or absorbed [25, 26]. This low bioavailability of iron from plant sources tends to increase the recommended iron intake in subjects following a vegetarian diet. Some authors suggest that vegetarians need to increase dietary iron by 80% to achieve normal serum iron and ferritin concentrations [27]. By contrast, the high consumption of fruit and vegetables by vegetarians results in higher dietary intake of vitamin C, which acts by reducing Fe^3+^ to the more soluble Fe^2+^ and improving dietary iron bioavailability. The vegetarians in our study had higher (by about 30%) intake of vitamin C in their diets than omnivores and this can partly counteract the inhibitory effects of high intakes of phytates or polyphenols on nonheme iron absorption. Generally, our group of vegetarian children was under nutritionist and medical care and followed balanced lacto-ovo vegetarian diet.

A range of hematologic and biochemical indicators can be used to assess iron status and to detect iron deficiency [1, 26]. In both our groups of children, the values of hemoglobin, MCV, and red blood cells were comparable. Among biochemical parameters, we observed similar mean values of serum transferrin and iron in both groups of children. Despite a normal mean value of serum iron, about 25% of vegetarians and 15% of omnivores had iron levels lower than 11 μmol/L. Serum ferritin concentrations were significantly decreased in vegetarians than omnivores, with a higher percentage of insufficiency in vegetarian group (25 vs. 5%, respectively). According to several studies, adults as well as children following vegetarian diets often have lower serum ferritin levels than omnivores, which is indicative of reduced iron stores, despite comparable iron intakes [16, 18, 23]. Similar to other authors, we did not observe differences in hematologic parameters between children on omnivorous and vegetarian diets.

To date, serum hepcidin has been mainly investigated in adult populations. The authors found that serum hepcidin levels in men remained constant over age and were higher than in premenopausal women. However, in postmenopausal women, the concentration of hepcidin increased [28]. In healthy pregnant women, concentrations of this hormone decreased gradually in the course of pregnancy to maximize iron absorption and availability, thus enhancing transfer to the fetus [8, 29]. There was no correlation between hepcidin in mothers and children, which may suggest that neonatal hepcidin is probably regulated independently of maternal hepcidin concentrations [30, 31].

There were two studies presenting hepcidin levels in healthy children [2, 3]. Weiler et al. [2] measured serum hepcidin concentrations in a multiethnic large group of preschool-age healthy children in Canada and found that hepcidin did not vary with any demographic variable. Our results were similar to Choi et al. [3], who determined the same bioactive form of hepcidin-25 in prepubertal children. We observed significantly lower hepcidin and higher sTfR concentrations in vegetarian children compared with peers on omnivorous diet. Comparable to others [3, 28], we found that serum hepcidin level was positively associated with ferritin and CRP concentrations in the whole group of children. Despite a correlation between hepcidin and CRP, there were no signs of inflammation in all the studied subjects, as CRP values were low.

We did not observe correlations between hepcidin concentration and iron status markers, anthropometric parameters, and dietary variables in omnivores; however, in vegetarians, we showed significant relations between hepcidin concentration and dietary iron and protein intakes. We also found a negative correlation between sTfR and ferritin in the whole group of children and positive correlation between sTfR and hemoglobin as well as red blood cells in omnivorous children. In the multivariable regression model, we confirmed the relationship between hepcidin and ferritin, but there was no association between sTfR and anthropometric, dietary and biochemical variables. Increased erythropoiesis in vegetarian children manifested by elevated sTfR concentration coexisting with decreased hepcidin levels might result from a subclinical iron deficiency in these children. The lower than sufficient ferritin levels observed in about 25% of our vegetarian subjects seem to confirm this hypothesis.

The presented work is the first study assessing serum hepcidin and soluble transferrin receptor levels in children on a vegetarian diet. Thus far, these parameters have been measured in children with diseases such as iron deficiency anemia, anorexia nervosa, and obesity. In children with iron deficiency and iron deficiency anemia, lower serum hepcidin and ferritin but higher sTfR were observed [2, 3]. In adolescents with anorexia nervosa, Pappilard-Marechal et al. [32] observed significantly higher serum ferritin and hepcidin levels than in healthy subjects. They found a highly significant correlation between ferritin and hepcidin concentrations and suggested that starvation in anorectic patients might stimulate ferritin and hepcidin gene expression. This hypothesis is also supported by the observation that serum ferritin and hepcidin levels return to normal after partial nutritional intervention.

Recently, hepcidin gene and protein expression were described in adipose tissue, with expression being higher in obese compared with lean subjects [33–37]. Aeberli et al. [34] and Hamza et al. [36] observed elevated levels of ferritin, hepcidin, and sTfR in obese children compared with normal weight peers, despite similar dietary iron intake. Additionally, weight loss in obese children is associated with decrease hepcidin levels and a significant improvement in iron absorption. Amato et al. [35] observed a direct correlation between hepcidin and leptin levels in children after weight loss therapy. The researchers suggested that in overweight children, iron availability is reduced for erythropoiesis and this is not due to low dietary iron but rather hepcidin-mediated reduced iron absorption and/or increased iron sequestration. However, hepcidin expression is modulated not only by body iron stores but also by hypoxia and inflammation. In addition, hepcidin is not only expressed in the liver but also in adipose tissue. Hence, leptin has been shown to stimulate hepcidin mRNA synthesis [33]. We previously reported a decrease in fat proportion and lower level of serum leptin in vegetarian children [38]. Our present results documented lower concentrations of hepcidin in children on a lacto-ovo vegetarian diet. It is possible that there may be an association between serum hepcidin and leptin levels.

Hepcidin is a relatively new marker for the assessment of iron status and there is still lack of therapeutic reference ranges. We observed, in multivariable analysis, positive correlation between ferritin (usual marker) and hepcidin (novel marker). Choi et al. [3] suggested that serum hepcidin concentration <6.895 ng/mL had sensitivity of 79.2% and specificity of 82.8% for diagnosing iron deficiency, and hepcidin <2.735 ng/mL had sensitivity of 88.1% and specificity of 88.2% for diagnosing iron deficiency anemia. Taking into consideration these criteria, concentration of hepcidin was below 6.894 ng/mL in 22 (51.2%) in our vegetarian children and in 8 (17.4%) omnivorous children. Compared with ferritin, which was lower in 23.3% of vegetarians and in 4.6% of omnivores, we suggested that hepcidin can be useful in detection of early stage of iron deficiency. Our results and results of other researchers will contribute to determining the cutoffs for hepcidin as an indicator in early stage of iron deficiency. sTfR is a biochemical parameter that is not cofounded by inflammation. Its levels are decreased in situations characterized by diminished erythropoietic activity and increased when erythropoiesis is stimulated by hemolysis or ineffective erythropoiesis. Elevated sTfR levels are also the characteristic feature of functional iron deficiency, a situation defined by tissue iron deficiency despite adequate iron stores. Some authors suggested that sTfR used in conjunction with ferritin (sTfR/ferritin index) may be better indicator than sTfR alone [39, 40]. In our study, serum concentrations of sTfR as well as the sTfR/ferritin index (0.063 vs. 0.04) were significantly higher (*p* < 0.001 and *p* < 0.05, respectively) in vegetarian than in omnivores. Therefore, we suggest that hepcidin as well as transferrin receptor might be useful in the assessment of iron metabolism in children (especially vegetarians), in order to prevent subclinical iron deficiency.

Our study had a few potential limitations. First, our sample size was relatively small, which lacks sufficient power to detect moderate associations with statistical significance. However, the whole group of investigated children was ethnically homogenous comparable in age and sex. Additionally, all vegetarians were following a lacto-ovo vegetarian diet. Second, we determined only CRP level, but did not measure other inflammatory markers, such as IL-6 (a particularly prominent inducer of hepcidin); however, all children were healthy with no signs of inflammation.

In conclusion, as hematologic parameters and iron concentrations in vegetarians and omnivores were comparable and ferritin level was lower in vegetarians, we suggest that inclusion of novel markers, in particular hepcidin and sTfR (not cofounded by inflammation), can better detect subclinical iron deficiency in children following vegetarian diets.
